# IL-6 Induced STAT3 Signalling Is Associated with the Proliferation of Human Muscle Satellite Cells Following Acute Muscle Damage

**DOI:** 10.1371/journal.pone.0017392

**Published:** 2011-03-09

**Authors:** Kyle G. Toth, Bryon R. McKay, Michael De Lisio, Jonathon P. Little, Mark A. Tarnopolsky, Gianni Parise

**Affiliations:** 1 Department of Kinesiology, McMaster University, Hamilton, Ontario, Canada; 2 Department of Pediatrics, McMaster University, Hamilton, Ontario, Canada; 3 Medical Physics and Applied Radiation Sciences, McMaster University, Hamilton, Ontario, Canada; University of Birmingham, United States of America

## Abstract

**Background:**

Although the satellite cell (SC) is a key regulator of muscle growth during development and muscle adaptation following exercise, the regulation of human muscle SC function remains largely unexplored. STAT3 signalling mediated via interleukin-6 (IL-6) has recently come to the forefront as a potential regulator of SC proliferation. The early response of the SC population in human muscle to muscle-lengthening contractions (MLC) as mediated by STAT3 has not been studied.

**Methodology/Principal Findings:**

Twelve male subjects (21±2 y; 83±12 kg) performed 300 maximal MLC of the quadriceps femoris at 180°•s^−1^ over a 55° range of motion with muscle samples (*vastus lateralis*) and blood samples (*antecubital* vein) taken prior to exercise (PRE), 1 hour (T1), 3 hours (T3) and 24 hours (T24) post-exercise. Cytoplasmic and nuclear fractions of muscle biopsies were purified and analyzed for total and phosphorylated STAT3 (p-STAT3) by western blot. p-STAT3 was detected in cytoplasmic fractions across the time course peaking at T24 (p<0.01 vs. PRE). Nuclear total and p-STAT3 were not detected at appreciable levels. However, immunohistochemical analysis revealed a progressive increase in the proportion of SCs expressing p-STAT3 with ∼60% of all SCs positive for p-STAT3 at T24 (p<0.001 vs. PRE). Additionally, cMyc, a STAT3 downstream gene, was significantly up-regulated in SCs at T24 versus PRE (p<0.05). Whole muscle mRNA analysis revealed induction of the STAT3 target genes *IL-6*, *SOCS3*, *cMyc* (peaking at T3, p<0.05), *IL-6Rα* and *GP130* (peaking at T24, p<0.05). In addition, *Myf5* mRNA was up-regulated at T24 (p<0.05) with no appreciable change in *MRF4* mRNA.

**Conclusions/Significant Findings:**

We demonstrate that IL-6 induction of STAT3 signaling occurred exclusively in the nuclei of SCs in response to MLC. An increase in the number of cMyc+ SCs indicated that human SCs were induced to proliferate under the control of STAT3 signaling.

## Introduction

Muscle satellite cells (SCs) are a population of cells that reside between the sarcolemma and basal lamina of myofibres [1] and have been shown to play an integral role in skeletal muscle repair [2], hypertrophy [3], [4], and hyperplasia [5], [6], in humans and animals. Although SCs are key regulators of muscle growth during development and muscle adaptation following exercise [7]–[15], the cellular regulation of human muscle SC function remains largely unexplored. Undoubtedly, the orchestration of events that govern SC function following damage involves a complex milieu of factors originating from the SC in addition to niche factors extrinsic to the SC [16]. Identified regulators of human SCs include insulin like growth factor-1 [17], hepatocyte growth factor [18], transforming growth factor beta [19] and Notch/Delta [7]. Recently, interleukin-6 (IL-6) has been implicated as playing a role in the regulation of human SCs in response to damaging eccentric contractions [20].

Traditionally, IL-6 was considered an inflammatory cytokine [21], however, recent work has shown that IL-6 is produced by muscle [22], released into circulation [23] and can act on the muscle cells themselves. As such, IL-6 is now also referred to as a “myokine” [24], [25]. Importantly, IL-6 knockout mice demonstrated a blunted hypertrophic response and less SC-mediated myonuclear accretion compared to wild-type mice following compensatory hypertrophy [26]. Furthermore, SCs from IL-6^−/−^ mice demonstrated an impaired proliferative capacity, both *in vivo* and *in vitro*, which was shown to be related to a lack of IL-6-mediated signal transducer and activator of transcription-3 (STAT3) signalling [26]. We have recently reported an increase in IL-6 protein localized in SCs 24 hours following a damaging bout of muscle-lengthening contractions (MLC) in humans coinciding with an increase in *cyclin D1* expression and SC number [20]. These data indicate that IL-6, acting via the janus kinase 2 (JAK2)/STAT3 signalling pathway, may be involved in SC proliferation/activation.

STAT3 is a downstream target of IL-6 [27], [28], and in response to IL-6 binding, STAT3 is phosphorylated via JAK2. This leads to the subsequent homodimerization and translocation of p-STAT3 to the nucleus [29]. Once in the nucleus, p-STAT3 binds to the γ-interferon activation sequence (GAS) element where it then promotes the transcription of downstream genes [30]. These genes have been shown to be responsible for a number of cellular functions including proliferation, migration, as well as anti-apoptotic functions [26]. *cMyc* is a downstream target gene in the STAT3 signalling cascade. It has been shown to regulate cell-cycle kinetics through the up-regulation of a number of Cyclin proteins which are involved in the cell growth phase G1 [31]–[33]. Furthermore, STAT3 also regulates a number of its upstream signalling cascade members including IL-6, GP130, IL-6Rα and suppressor of cytokine signalling 3 (SOCS3). The STAT3 pathway is regulated in a negative feedback loop through interactions with JAK2 [34]. SOCS3 can bind phosphotyrosines on JAK2 and physically block STAT3 from binding to JAK2. Additionally, SOCS3 can recruit ubiquitin-transferases leading to the ubiquitination and degradation of JAK2 [29].

Based on previous work by McKay and colleagues (2009) showing that p-STAT3 co-localized with SCs we aimed to quantify SC localized p-STAT3 signalling over a time course. We hypothesized that the mRNA species of the IL-6/STAT3 signalling cascade would be up-regulated early following the MLC protocol along with a similar increase, as reported previously by McKay and colleagues (2009), in the IL-6^+^/Pax7^+^ cell population. Furthermore, using a time course directed at capturing STAT3 phosphorylation, we hypothesized that we would observe an increase in p-STAT3 specifically in the SC population coinciding with an increase in SC number. In addition, we hypothesized that cMyc, a downstream product of STAT3 signalling, would be detected with mRNA up-regulated in whole muscle and protein co-localized to SCs following an increase in STAT3 signalling in the SC population.

## Results

**Figure 1:**
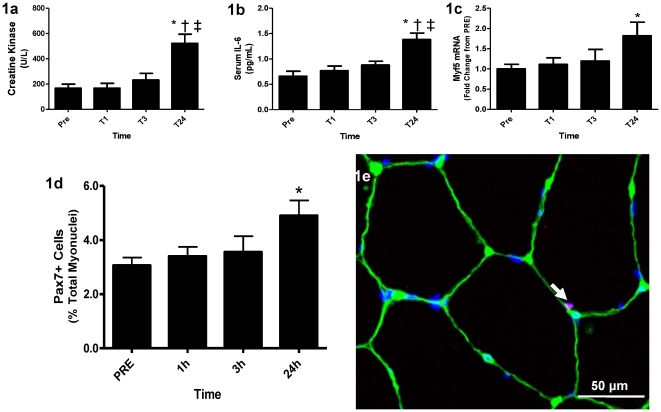
Serum measures and Pax7 positivity. (**1a**) Average serum CK response in U/L. (**1b**) Average serum IL-6 response in pg/mL; note the similar serum responses between IL-6 and CK. (**1c**) Myf5 mRNA expression relative to GAPDH, expressed as fold change from PRE. (**1d**) Pax7^+^ cells as a percentage of total myonuclei over the time-course. (**1e**) Representative image at 40× magnification of a Pax7/Laminin stain with Pax7 in red, Laminin in green and DAPI in blue. Values are reported as mean ± S.E.M. *p<0.05 vs. PRE; † p<0.05 vs. T1, ‡ vs. T3.

To confirm that the MLC protocol caused muscle damage we examined serum creatine kinase (CK) levels over the time course. Serum CK peaked 24 hours post MLC, increasing over 300% from PRE levels (p<0.05 vs. PRE) (figure 1a). Serum IL-6 also peaked 24 hours post-MLC (p>0.05 vs. PRE), as well, increasing over 200% from PRE (figure 1b). These measures were correlated indicating a relationship between muscle damage and serum IL-6 (R^2^ = 0.3055; p<0.001) (figure S1a)

In response to acute muscle damage we observed a 26.6% increase in Pax7^+^ cells 24 hours following the MLC protocol. Pax7^+^ cells per 100 myofibers increased from 15.5 at PRE to 19.6 (p<0.05) 24 hours post exercise. When expressed as a percentage of total myonuclei we observed a 60.3% increase in satellite cell number (∼3% at PRE to ∼4.5% 24 hours post) (p>0.05 (figure 1d)). Satellite cells were quantified using a Pax7/Laminin co-stain (figure 1e) to ensure that all Pax7 cells were in the SC niche. Furthermore, myogenic regulatory factor 5 (*Myf5*), known for its role in SC proliferation was significantly up-regulated 1.8 fold 24 hours following the MLC (p<0.05) (figure 1c), while no significant change was observed for myogenic regulatory factor 4 (*MRF4)* (data not shown), known for its role in differentiation.

**Figure 2:**
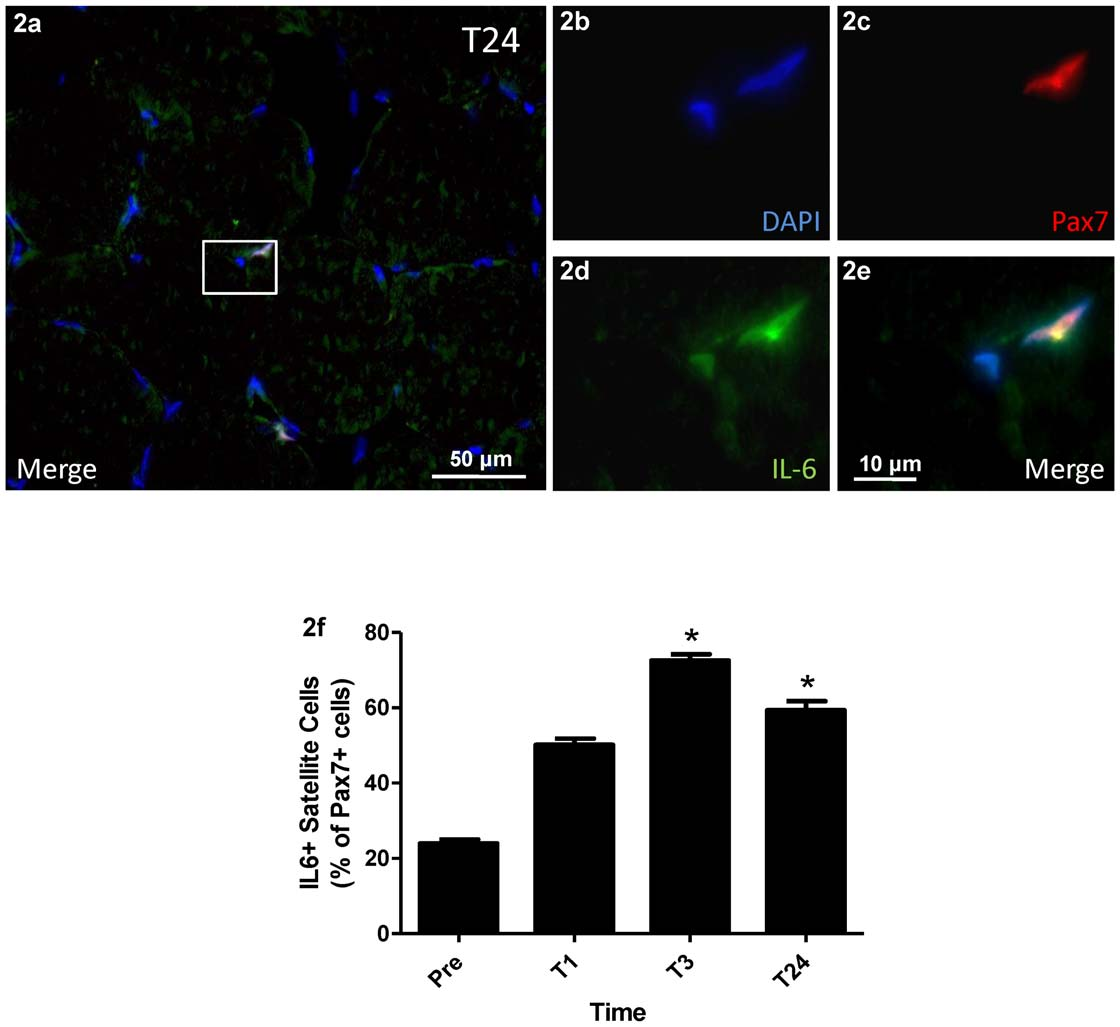
IL-6/Pax7 co-localization. (**2a**) Representative merged image of a muscle cross-section at 40× magnification stained for IL-6 in green, Pax7 in red, and nuclei in blue being DAPI^+^. The scale bar is 50 µm. (**2b–e**) **100×** magnification image of the inset box in 2a. Scale bar is 10 µm (**2b**) DAPI^+^ nuclei. (**2c**) Pax7^+^ nuclei. Note only one of the nuclei is Pax7 positive showing the specificity of the Pax7 stain (**2d**) IL-6^+^ nuclei. Note that both nuclei are IL-6 positive while there is less intense IL-6 staining in the fiber itself. (**2e**) Merged image showing the co-localization of DAPI, IL-6 and Pax7. (**2f**) IL-6^+^ cells as a percentage of Pax7^+^ cells. Values are reported as mean ± S.E.M. *p<0.05 vs. PRE.

The number of SCs expressing IL-6 was quantified (figure 2a–d). While there were relatively few co-positive SCs PRE, there was a significant increase in IL-6^+^/Pax7^+^ cells at 3 and 24 hours post MLC vs. PRE (p<0.05) with the peak observed at 3 hours post where 75% of Pax7^+^ cells were co-positive for IL-6 (figure 2e).

**Figure 3:**
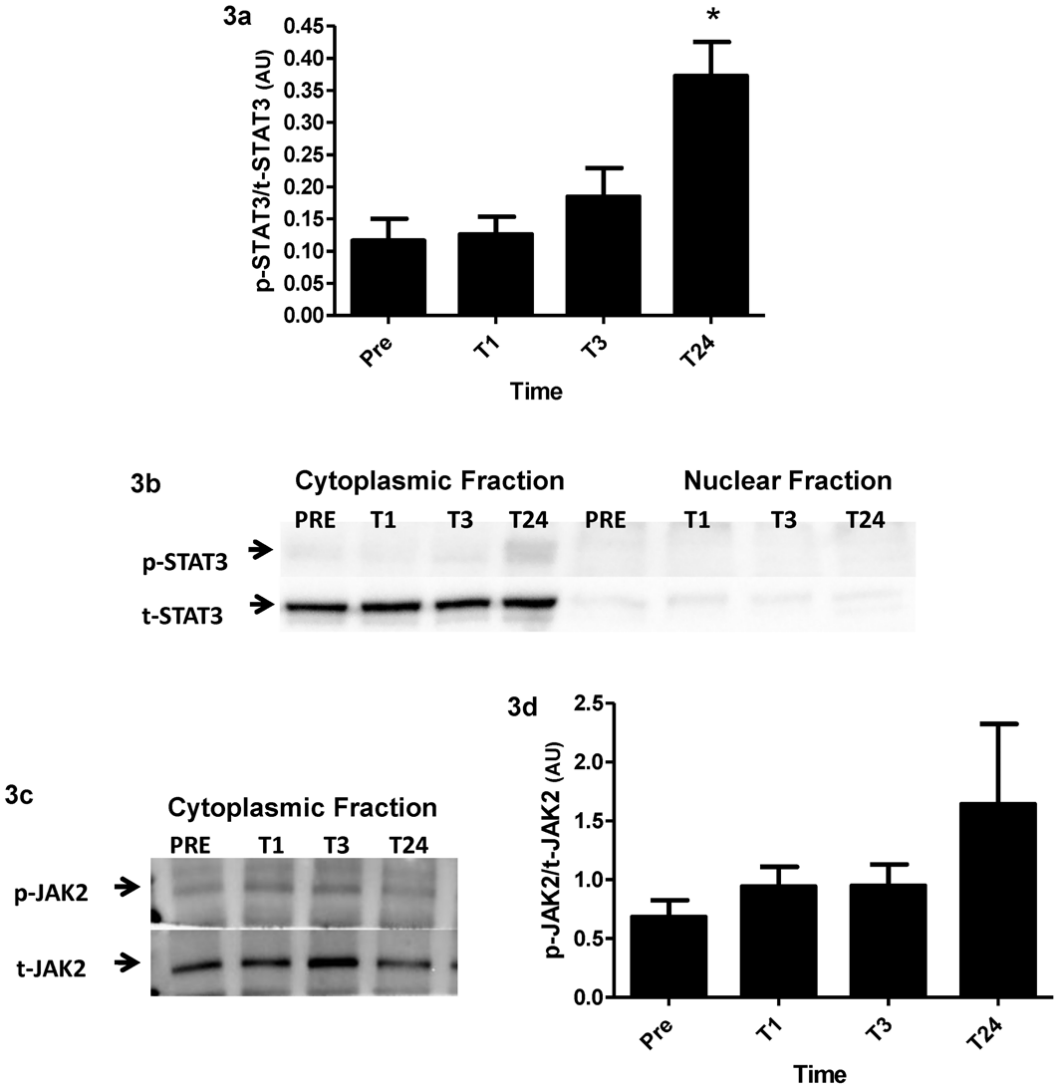
Nuclear and cytoplasmic expression of STAT3 and JAk2. (**3a**) Ratio of phosphorylated to total STAT3 protein in the cytoplasmic fraction. (**3b**) Representative images of p-STAT3 and t-STAT3 in both the cytoplasmic and the nuclear fraction. (**3c**) Representative images of p-JAK2 and t-JAK2 in the cytoplasmic fraction. (**3d**) Ratio of phosphorylated to total JAK2 in the cytoplasmic fraction. Values are reported as mean ± S.E.M. *p<0.05 vs. PRE.

Previous attempts to quantify the STAT3 response did not show a significant change at the whole muscle level, thus the nuclear and cytoplasmic fraction of whole muscle was examined for a more specific analysis. Purity was assessed using lactate dehydrogenase (LDH) as a nuclear marker and p84 as a cytoplasmic marker (figure S1b). Cytoplasmic p-STAT3 was significantly up-regulated 24 hours vs. PRE (p<0.05) when measured against t-STAT3 which remained unchanged over the time course (figure 3a). When the nuclear fractions were analysed; however, no detectable p-STAT3 and only traces of t-STAT3 were observed (figure 3b). JAK2 was also analyzed in the cytoplasmic fraction (figure 3c). The ratio of p-JAK2 to t-JAK2 was not significantly different at any time point (figure 3d).

**Figure 4:**
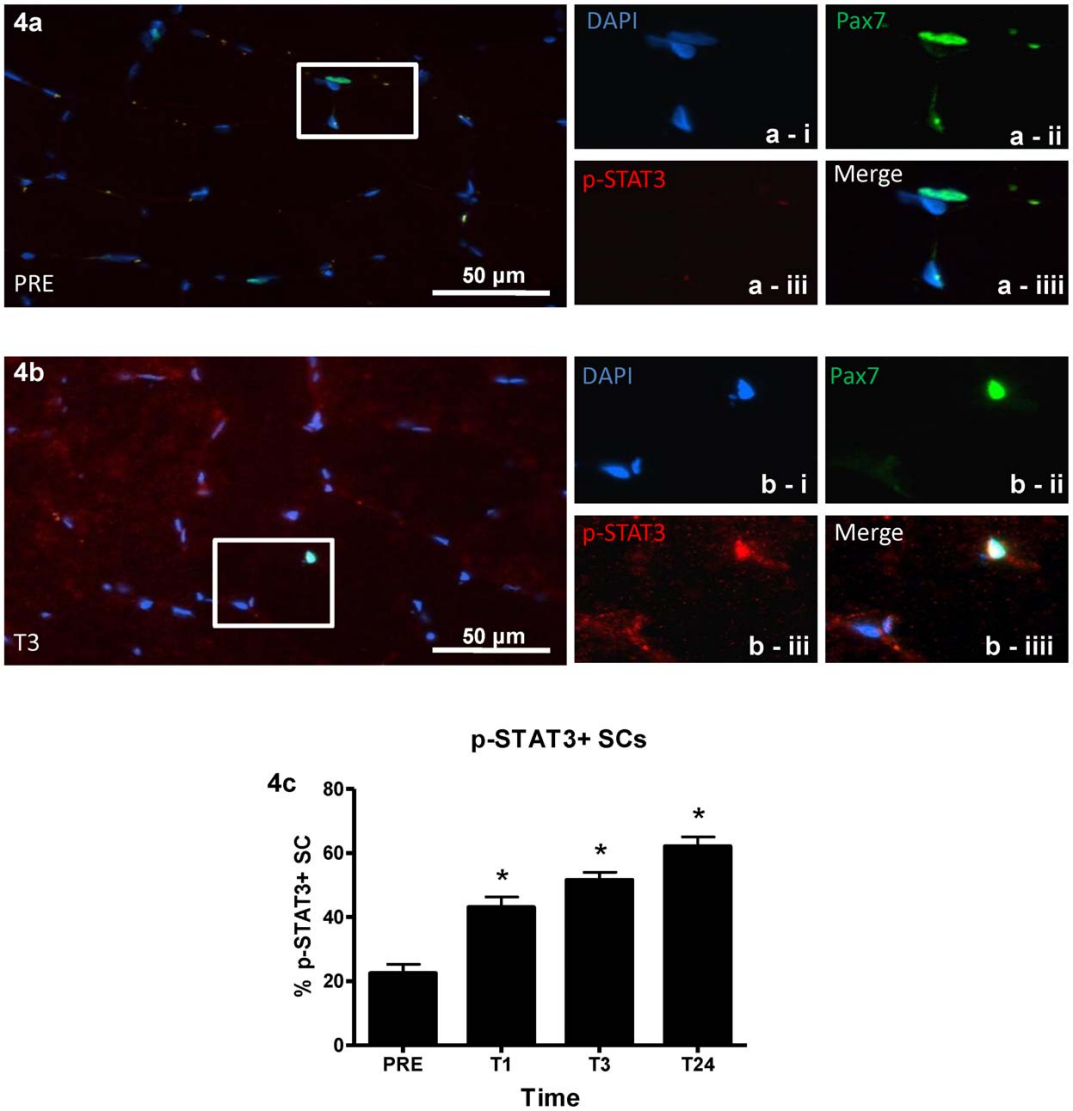
p-STAT3^+^/Pax7^+^ cells. (**4a**) Representative merged image of PRE at 40× magnification with inset box showing (**a-i**) DAPI^+^ nuclei, (**a-ii**) Pax7^+^ nuclei, (**a-iii**) no p-STAT3 stained nuclei and (**c-iiii**) a merged image. (**4b**) Representatvie merged image of T3 at 40× magnification with inset box showing (**b-i**) DAPI^+^ nuclei, (**b-ii**) Pax7^+^ nuclei, (**b-iii**) p-STAT3^+^ nuclei and (**b-iiii**) a merged image showing co-localiztion. Note that punctate p-STAT3 is not present at PRE but co-localizes with Pax7 at T3 and that there is an increase in diffuse fiber staining that occurs from PRE to T3. (**4c**) Percentage of p-STAT3^+^ SC as quantified over the time course peaking at T24. Values are reported as mean ± S.E.M. *p<0.05 vs. PRE.

**Figure 5:**
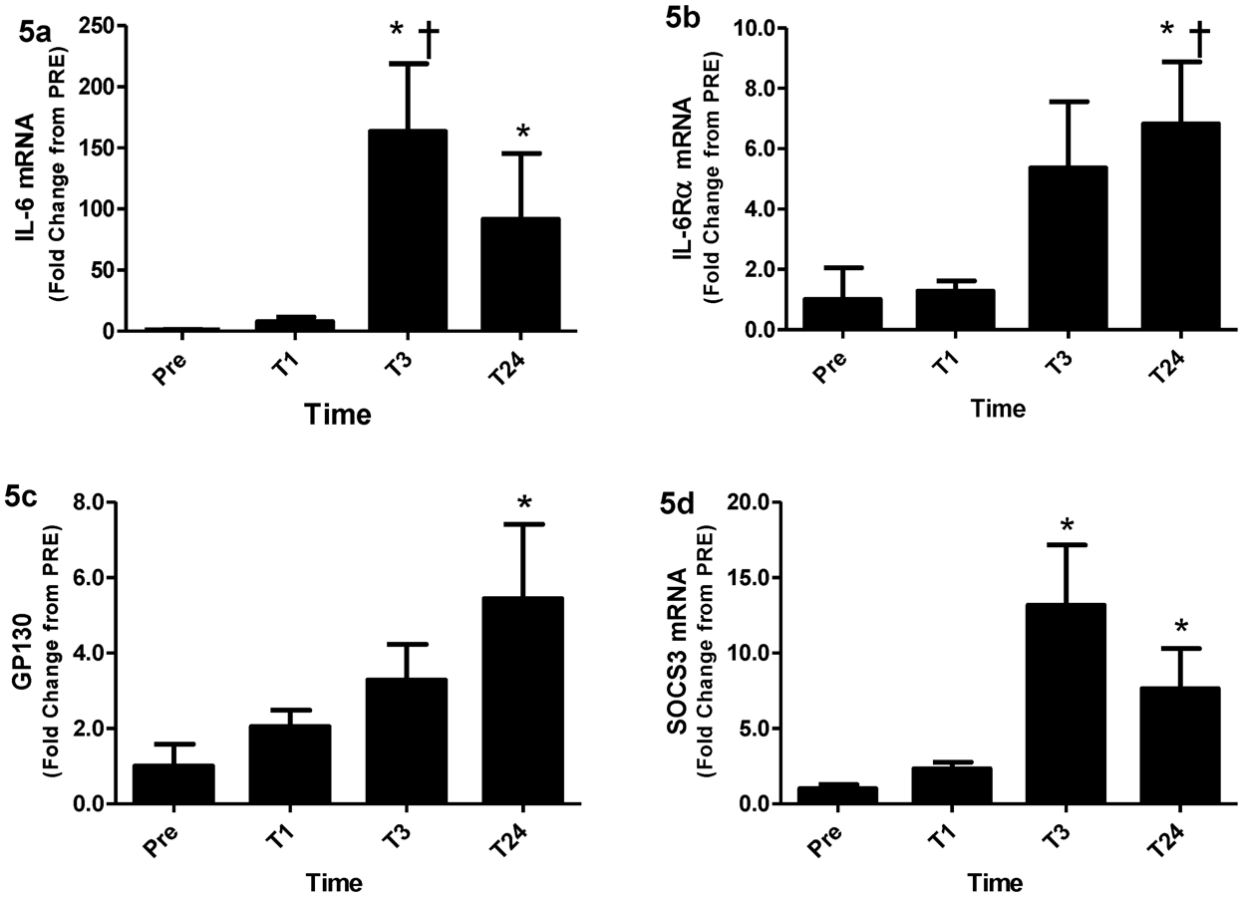
STAT3 downstream genes. (**5a**) IL-6, (**5b**) IL-6Rα, (**5c**) GP130 and (**5d**) SOCS3 mRNA expression relative to GAPDH, expressed as fold change from PRE. Values are reported as mean ± S.E.M. *p<0.05 vs. PRE; † p<0.05 vs. T1, ‡ vs. T3.

**Figure 6:**
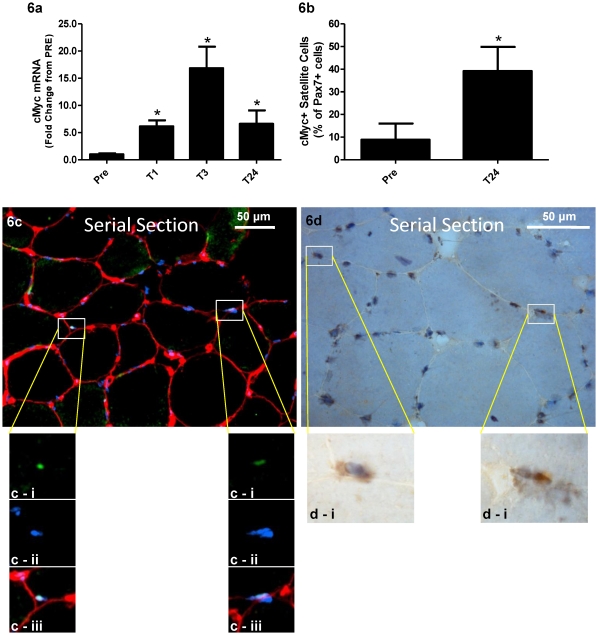
cMyc^+^/Pax7^+^ quantification at Pre and T24. (**6a**) cMyc mRNA relative to GAPDH, expression represented as fold changes from PRE. (**6b**) cMyc^+^ SC as a percentage of Pax7^+^ cells between Pre and T24, n = 7. (**6c & 6d**) Serial sections of the same muscle cross-section with higher magnification (100×) boxes showing the same nuclei in both images. (**6c**) Representative image at 20× magnification of a merged Pax7/Laminin costain where (**c-i**) Pax7 is in green, (**c-ii**) DAPI^+^ nuclei are in blue and (**c-iii**) laminin in red. Note the two Pax7^+^ nuclei which have been highlighted to show their individual Pax7 positivity, DAPI staining and location within the laminin boarder. These nuclei are highlighted again in (**6d**) where the same cells have been stained colourometrically for cMyc. (d-i) A magnified image of the inset box showing the brown cMyc staining co-localized to the nuclear marker hematoxalin The brown cMyc positive cells are clearly within the visible fiber boarder. *p<0.05 vs. PRE.

Although changes in p-STAT3 were not detectable in the enriched nuclear fractions via western blot, immunofluorescent analysis of p-STAT3 (figure 4a–b) illustrated an increase in p-STAT3^+^/Pax7^+^ cells as a percentage of total Pax7^+^ cells ranging from 20% PRE to 40% at 1 hour, 50% at 3 hours, and 60% at 24 hours (all significantly different vs. PRE p<0.05) (figure 4c). Diffuse p-STAT3 staining was observed throughout the muscle fibres and appeared to intensify over the time course (figure 4a–b), which agreed with the increased cytoplasmic p-STAT3 at 24 hours post observed using western blot analysis. Importantly, the diffuse p-STAT3 observed in the fibre almost never co-localized with non-satellite cell myonuclei. To further verify that p-STAT3 signalling was indeed occurring, downstream target genes of STAT3 were analyzed. *IL-6* mRNA peaked at 3 hours, up approximately 150 fold from PRE (p<0.05) and remained elevated, up ∼80 fold from PRE (p<0.05) at 24 hours (figure 5a). Both *IL-6Rα* (figure 5b) and *GP130* (figure 5c) mRNA showed significant increases peaking at 24 hours up 7 fold from PRE (p<0.05) and 4.5 fold from PRE (p<0.05) respectively. *SOCS3* also increased, peaking at 3 hours, up 13 fold (p<0.05) and remaining elevated at 24 hours up 8 fold (p<0.05) (figure 5d) from PRE. Furthermore, the expression of *SOCS3* was positively correlated (R^2^ = 0.5984, p<0.001) with the expression of *IL-6* across time (figure S1c). The cell-cycle related gene *cMyc* was robustly up-regulated from PRE over the entire time course peaking at 4 hours up 15 fold (p<0.05) (figure 6a). *cMyc* mRNA was positively correlated with *IL-6* mRNA (R^2^ = 0.2876, p<0.001) (figure S1d) and *SOCS3* mRNA (R^2^ = 0.5406, p<0.001) (figure S1e) illustrating a positive relationship in the temporal expression of these STAT3 target genes.

To further verify the mRNA data illustrating an increased expression of *cMyc*, immunohistochemical analysis of cMyc was coupled with Pax7 in serial sections (figure 6c–d) and the number of cMyc^+^/Pax7^+^ cells was quantified PRE and 24 hours following MLC. The percentage of cMyc^+^/Pax7^+^ cells was significantly increased at 24 hours (∼40%) versus PRE (∼9%) (figure 6b). This provides additional evidence that the STAT3 signalling cascade was active as a consequence of the bout of MLC in SCs leading to proliferation of the SC population.

## Discussion

Cell proliferation is a complex process regulated by a number of factors, such as IL-6, IGF-1, HGF and TNFα [17], [35], [26], [18], [36], among others. It is a process vital for continued functional capacity and overall tissue survival of skeletal muscle. Skeletal muscle fibres are post-mitotic thus the satellite cell (SC) is the exclusive source of new nuclei for the maintenance of healthy skeletal muscle. Here we illustrate the potential of STAT3 signalling in promoting SC proliferation following acute muscle damage in humans. We have demonstrated that following muscle damage, phosphorylated STAT3 (p-STAT3) in SCs increases early (within one hour), inducing downstream target genes (i.e. *GP130* and *SOCS3*), which further regulate the increase in STAT3 production and response (as induced via IL-6), leading to increased *cMyc* expression, which drives cell proliferation. As SCs account for such a small percentage of total myonuclei (∼2–7%), even when the nuclear fraction of whole muscle homogenate was analyzed, no p-STAT3 was observed. This lack of observable p-STAT3 in the nuclear fraction combined with the increase in p-STAT3^+^/Pax7^+^ cells, measured via immunohistochemistry strongly support the premise that p-STAT3 signalling occurred almost exclusively in SCs in human muscle. Further evidence is provided by the expansion of the cMyc^+^/Pax7^+^ cell population from PRE to 24 hours expressed as a percentage of total Pax7^+^ cells. Collectively, the time-course utilized in this study was successful in capturing a rapid up-regulation in p-STAT3 signalling in SCs shown to be associated with an increase in SC number following exercise induced muscle injury.

In agreement with previous studies [20], IL-6 mRNA and protein in both the blood and co-localized to the SC increased in response to MLC. The ∼1.5 fold increase in serum IL-6 supports a number of studies reporting that IL-6 is released from the muscle into circulation in response to MLC [20], [25], [37]. *IL-6* mRNA was significantly up-regulated 3 hours and 24 hours post MLC, up ∼150 fold and ∼100 fold respectively. While the fold changes were of a greater magnitude in this study than in McKay and colleagues (2009), most likely due to the dietary controls and the training status of the subjects, the directional temporal pattern of expression was similar between the two studies. Finally, IL-6^+^/Pax7^+^ cells increased from PRE, where there were relatively few co-positive cells, to a peak at 3 hours where approximately 75% of all SCs were IL-6^+^. IL-6^+^/Pax7^+^ cells remained significantly up-regulated from PRE at 24 hours, with approximately 60% of Pax7^+^ cells expressing IL-6. It is known that SCs express the IL-6Rα [38], [20] allowing for IL-6 signalling to occur. Thus, an increase in SC mediated IL-6 synthesis suggests that IL-6 signalling occurs in an autocrine/paracrine fashion. As a consequence, we suggest that increased IL-6 signalling leads to the induction of p-STAT3 signalling in the SC as evidenced by the increases in the p-STAT3^+^/Pax7^+^ population and the up-regulation of p-STAT3 regulated genes.

Previously p-STAT3 was found to be localized in the SC compartment; however, quantification of p-STAT3 over a time-course was not conducted in that study [20]. To verify that p-STAT3 regulated the SC response, we quantified p-STAT3 immunofluorescence. The proportion of p-STAT3^+^ SCs was significantly elevated at T1 with ∼40% of SCs co-positive and peaking at T24 where ∼60% of SCs were co-positive, similar to that of IL-6^+^ SCs. Importantly, this early increase in p-STAT3^+^ SCs verifies the rapid signalling of this system and confirms that the timing of these events were indeed occurring early as proposed by McKay et al. [20]. This provides temporal evidence that following a bout of damaging exercise, STAT3 is phosphorylated leading to downstream signalling events resulting in proliferation of human SCs. The significant increase in Pax7^+^ cells, the up-regulation of *Myf5* mRNA (a major regulator of SC proliferation) coupled with no significant change in *MRF4* mRNA (a major regulator of SC differentiation) and an increase in the percentage of cMyc^+^ SCs 24 hours after the MLC provide further evidence that human SC proliferation involved p-STAT3 signalling. In addition, because almost no other nuclei other than SCs were positive for p-STAT3 and the nuclear fraction western blots detected virtually no p-STAT3 as they were not sensitive enough to detect such a small percentage of cells expressing p-STAT3protein, it would appear that the IL-6 induced STAT3 signalling cascade occurred almost exclusively in SCs. This supports the observational report from McKay and colleagues who found that p-STAT3 was co-localized to SCs 4 hours after subjects had preformed 300 MLC [20] and work done by Kami & Senba who co-localized p-STAT3 to c-Met^+^ cells in rats following freeze crush injury [39]. Trenerry and colleagues also showed a transient response in myonuclei expressing p-STAT3 2 hours following leg extension exercise [28], however no SC specific analysis was conducted in that study.

It is also worth noting that there was a progressive increase in p-STAT3 staining in the muscle fibers over the time-course. This agrees with the stepwise increase in p-STAT3 observed in the cytoplasmic fraction. This may be explained by additional cellular functions of p-STAT3. p-STAT3 has been implicated in microtubule remodelling, matrix metalloproteinase production [40], [41], and focal adhesion protein production [42], [41], leading to increased migratory potential of different cell types such as inflammatory and SCs. In addition, p-STAT has also been shown to play a role in regulating the general hypertrophic response through its interactions with the leptin receptor in skeletal muscle [43], and angiotensin II in cardiac muscle [44]. Possibly, in response to muscle fiber damage, STAT3 is phosphorylated in damaged myofibers acting as a localized chemotactic stimuli for the SC and inflammatory cell populations to migrate toward. This allows for expeditious repair via phagocytosis of necrotic or damaged tissues [45], breakdown of connective tissues through matrix metalloproteinase activity [46] and efficient migration of SCs to the areas of damage [47].

There was no significant increase in p-JAK2 protein at any of the time points. As an upstream regulator of STAT3 phosphorylation we would expect a temporal profile for JAK2 phosphorylation similar to that of STAT3. Since cytoplasmic p-STAT3 peaked at 24 hours following MLC it is possible that we missed the increase in p-JAK2 as it may have occurred sometime between 3 and 24 hours.

In addition to the increase in serum IL-6 and *IL-6* mRNA, we also observed an up-regulation of several STAT3-related downstream genes. *SOCS3* expression, downstream of STAT3 signalling serves as a negative regulator of JAK/STAT signalling. *SOCS3* functions via a classic feedback inhibition loop. Production of SOCS3 is up-regulated in response to JAK/STAT signalling whereby it then acts to suppress JAK/STAT signalling by inhibiting the activity of JAK2 kinase [34], [29]. Importantly, the temporal expression of SOCS3 was closely related to the temporal expression of IL-6 with a peak at 3 hours and remaining elevated at 24 hours post MLC. *cMyc*, a gene known to promote proliferation, and that is expressed downstream of STAT3 [32], was also associated with the same pattern of temporal expression as IL-6 and SOCS3 following MLC, as demonstrated through significant correlations with each of these genes (figures S1d–e). Two other necessary members of this signalling cascade are the receptor sub-complexes *IL-6Rα* and *GP130*. They both revealed increases in their mRNA expression over the time course with a peak at 24 hours. This is important as both of these mRNA species are up-regulated in response to STAT3 signalling and serve to autoregulate the pathway.

Providing yet another level of support for STAT3 mediated SC proliferation was the increase in the number of cMyc^+^/Pax7^+^ cells observed at T24 versus PRE. Previous work by McKay and colleagues [20] did not localize the downstream signalling events of the STAT3 pathway to the SC, looking only at different mRNA species in whole muscle. Importantly, this work definitively shows that the change observed in *cMyc* mRNA translates into significant protein expression specifically in the SCs during a time when these cells were proliferating. This implicates STAT3 as a primary inducer of SC proliferation in human muscle following MLC. The cMyc^+^/Pax7^+^ population was only analysed PRE and 24 hours post MLC as this was the time when the highest levels of proliferation were occurring as evidenced by the significant increase in Pax7^+^ cells. cMyc is a critical regulator of the cell cycle, responsible for the transition from G1 to S phase [48]. It has also been shown to be up-regulated in response to IL-6 signalling [49] and in a STAT3 induced GP130 mediated manner [32]. One of its main downstream functions is to regulate the production of CyclinD1, a known regulator of the cell cycle, which we have previously reported to increase in response MLC. The increase in SCs expressing cMyc serve as direct evidence that p-STAT3 signalling is occurring in SCs leading to their proliferation.

The STAT3 signalling cascade as induced by IL-6 has been shown here to be an important regulator for the proliferation of SCs early following a muscle damaging protocol. Collectively, we have shown that SCs up-regulate IL-6 following damage which likely acts in an autocrine/paracrine fashion to promote proliferation through STAT3 signalling. This IL-6 induced STAT3 signalling is evidenced by the up-regulation of the downstream genes regulated by p-STAT3. Furthermore, with p-STAT3 being observed almost exclusively in SCs, not in other myonuclei and only to a large degree at least 1 hour post muscle damage, it appears that it acts in a very specific manner through IL-6 leading to the proliferation of SCs and subsequent repair of muscle damage. Thus, as evidenced by the induction of cMyc protein it appears that STAT3 is a key signalling molecule in human SCs in response to physiological levels of muscle damage and contributes to the robust proliferation of SCs in the acute period following damage.

## Materials and Methods

### Ethics Statement

All subjects were informed of the procedures and the potential risks associated with the study and gave written informed consent. This study was approved by the Hamilton Health Sciences Research Ethics Board (08-413) and conformed to the Declaration of Helsinki regarding the use of human subjects as research participants.

### Subjects

Twelve healthy males (age 21.2±1.6 yrs, height 178.2±5.5 cm and weight 82.6±11.5 kg) were recruited from the McMaster University community. Subjects were sedentary having done no lower body resistance exercise for at least the past 6 months and were non-smokers. Subjects were told to refrain from doing any moderate or strenuous exercise for two days prior to and during the study.

### Muscle Damage Protocol

The muscle damage protocol was preformed as described previously [50]. Briefly, subjects completed 300 unilateral isokinetic eccentric contractions of the *quadraceps femoris* using a Biodex dynamometer (Biodex-System 3, Biodex Medical Systems, Inc., Shirley USA) at 180°•s^−1^ over a 55° range of motion [20], [17], [18]. The experimental leg was chosen as the dominant leg. During each set investigators provided verbal encouragement so as to elicit maximal effort from the subjects. The entire duration of the protocol was approximately 30 min of which the muscle was under tension for about 10 min.

The protocol employed in the present study has previously been shown to cause physiological muscle damage as evidenced by increased serum creatine kinase (CK) [50]–[52], z-band streaming [53], [54], desmin disruption, a significant infiltration of the inflammatory related macrophages and neutrophils, as well as a significant myogenic response leading to an increase in satellite cell number based on both Pax7 and NCAM staining [20], [17], [18].

### Muscle Biopsies

Subjects reported to the lab at either 7, 7:30, or 8am having completed an overnight fast. Upon arrival to the lab subjects rested, had their PRE blood draw taken from the *antecubital vein* and then underwent the muscle damaging protocol. Once the subjects had completed the protocol they had one hour to rest. One hour post muscle damage the subjects had a second blood draw taken as well as two muscle biopsies; one from the exercise leg (T1) and one from the control leg (PRE). Three hours after the protocol the subjects had a third blood draw taken along with another biopsy from their exercise leg (T3). Subjects were then given a meal replacement beverage (Ensure, Abbott Laboratories, Abbott Park, Illinois, U.S.A.) The subjects were instructed to refrain from taking any anti-inflammatory drugs or doing any vigorous exercise. The subjects arrived the next morning at 8, 8:30, or 9am in a fasted state for the final (T24) blood draw and biopsy from the exercised leg.

Muscle biopsies were obtained using the percutaneous needle [55], [56] method using manual suction from the vastus lateralis under local anaesthetic (1% lidocaine) as described previously [57]. In total, four muscle biopsies were obtained from each subject, three in the exercise leg and one in the control leg (non-exercise leg). The muscle tissue was dissected free of adipose and connective tissue. The tissue was immediately divided into four sections for RNA and protein (western blotting and immunohistochemical) analysis, three of which were flash-frozen in liquid nitrogen and stored at −80°C for later analysis and the final piece embedded in Optimal Cutting Temperature (OCT) compound embedding medium and frozen in liquid nitrogen cooled isopentane.

### Blood Measures

Blood samples were obtained from the antecubital vein immediately prior to the intervention and then again concurrently with the muscle biopsies one, three, and twenty-four hours after the muscle damage protocol. Approximately 8 mL of blood was taken from each subject at each time point. 4 mL was drawn into a heparinised tube while the other 4 mL was drawn into a non-heparinized tube to obtain plasma and serum samples respectively. Samples were allowed to sit on ice (plasma) or at room temperature (serum) for 15 minutes, centrifuged at 4000 RPM for 15 minutes, aliquoted into 600 µL aliquots and frozen down at −80°C for later analysis.

Serum analysis was conducted for both creatine kinase activity and IL-6 protein. Creatine kinase activity was analysed using a commercially available kit (Pointe Scientific, Inc., Canton USA) with modifications to the protocol to allow for running the samples at 25°C. This included adding additional sample using a ratio obtained from the International Federation of Clinical Chemistry (IFCC) for conversion between 37°C and 25°C. Additionally, sample absorbance was measured every twenty seconds for twenty minutes to obtain the slope of the change in absorbance/minute. This value was used to calculate the concentration of creatine kinase in international units (U/L) which is defined as the amount of enzyme that catalyzes the transformation of one micromole of substrate per minute.

Serum IL-6 was analysed using a commercially available high sensitivity Quantikine Enzyme-Linked ImmunoSorbent Assay (ELISA) kit according to the manufacturer's instructions (R&D systems Inc., Minneapolis USA). Samples were run in duplicate with all subjects on the same plate and an intra-assay CV of 6.9%.

### RNA Isolation

RNA was isolated was conducted as previously described [20]. RNA isolation was conducted using Trizol Reagent (Invitrogen Corporation, Carlsbad USA) and RNA purification was done using the Qiagen RNeasy mini kit (Qiagen Sciences, Mississauga Canada). The RNA was then quantified using a spectrophotometer (NanoDrop 1000, Thermo Fisher Scientific Inc., Wilmington USA).

### Reverse Transcription (RT)

RNA was transcribed to cDNA using Applied Biosciences High Capacity cDNA reverse Transcription Kit (Applied Bioscience, Foster City USA). Individual samples in 20 µL reactions were reverse transcribed into cDNA using an Eppendorf Mastercycle epigradient thermal cycler (Eppendorf, Mississauga Canada).

### Quantitative Real-Time Polymerase Chain-Reaction (qRT-PCR)

qRT-PCR was performed in 25 µL reactions using SYBR Green/Rox master mix (SuperArray Bioscience Corp., Frederick USA). Primers were custom made using published sequences (table S1). They were resuspened in 1× TE buffer (10 mM Tris-HCL, 0.11 mM EDTA) and frozen at −20°C until use. Using 0.2 mL PCR tubes (Axygen Inc., Union City USA) 12.5 µL of SYBR green, 2 µL forward primer, 2 µL reverse primer, 6.5 µL or 7.5 µL of H_2_O depending on the cDNA template volume, 1 µL or 2 µL was added depending on the amount necessary for the particular gene of interest (25 ng cDNA or 50 ng cDNA). qRT-PCR was performed using a Stratagene Mx3000P real-time PCR system (Stratagene, Santa Clara USA) and Stratagene MXPro QPCR Software Version 3.00 (Stratagene, Santa Clara USA). Changes in gene expression over time were expressed as fold changes ± SEM from pre values using the delta delta CT method [58] with glyceraldehyde 3-phosphate dehydrogenase (GAPDH) as a housekeeping gene. GAPDH expression was not different from PRE at any of the post-intervention time-points.

### Nuclear and Cytoplasmic Extraction

Nuclear and cytoplasmic extraction was performed using a commercially available kit with minor modifications (NE-PER Nuclear and Cytoplasmic Extraction Reagents – Thermo Fischer Scientific Inc., Wilmington USA). Between 20 mg and 60 mg of tissue were transferred into a 2 mL microcentrifuge tube while still frozen. 10× the volume in µL of the weight of the sample was added to the sample for CER I buffer (from kit with the addition of one protease and one phosphatase tablet dissolved in it). The muscle was then minced using mincing scissors in the 2 mL microcentrifuge tube for about 15 seconds. The sample was then homogenized four times for five seconds using a rotary homogenizer (PRO250, PRO Scientific Inc., Oxford USA). The homogenate was then transferred to a 1.5 mL microcentrifuge tube and vortexed for fifteen seconds. Following vortexing, 20 µL of CER II buffer was added per sample (from kit with the addition of half a protease and half a phosphatase tablet dissolved). The sample was then vortexed for five seconds and put on ice for one minute. The tube was again vortexed for five seconds and then centrifuged for five minutes at 16 000 g at 4°C. The supernatant (cytoplasmic extract) was immediately transferred to pre-chilled 1.5 mL microcentrifuge tubes and frozen down at −80°C. The remaining pellet was then washed with 400 µL of a PBS cocktail containing one protease and one phosphatase tablet dissolved, spun for five minutes at 16 000 g at 4°C. The liquid was decanted with the wash repeated three more times. On the last wash the pellet was resuspended prior to the final spin. After the final spin the pellet was suspended in 200 µL of ice-cold NER buffer (from kit with the addition of 50 µL of 10% SDS). The pellet was then broken up using Teflon pestles. The samples were then vortexed for 15 seconds and placed on ice for ten minutes. This was repeated three more times. The samples were then centrifuged at 16 000 g for ten minutes at 4°C. The supernatant (nuclear extract) was then immediately transferred to a pre-chilled 1.5 mL microcentrifuge tube and frozen down at −80°C. Bradford analysis was then conducted on the samples to obtain the concentration using a spectrophotometer (UltraSpec 300 pro, Biochrome Ltd., Cambridge UK).

### Western Blotting

Equal amounts (75 µg) of cytoplasmic or nuclear homogenate in 4× Laemlli buffer were boiled at 95°C for 5 minutes then loaded in the wells of at 7.5% gel. Nuclear samples were precipitated via the acetone precipitation [59] method to increase their concentrations so that 75 µg of nuclear protein could be loaded in each well. Briefly, samples were mixed with four volumes of ice cold acetone and incubated for 1 hour. Samples were spun at 13,000 g for 10 minutes and the resulting pellet was dried for 20 minutes. Dried pellets were reconstituted in 4× Laemlli buffer and ddH_2_O. Phosphorylated and total Jak2 were analyzed in the cytoplasm only, while phosphorylated and total Stat3 were analyzed in both the nucleus and cytoplasm. Nuclear p-STAT3 was run with cytoplasmic pSTAT3 from the same subject. Gels were run at 125 V for approximately 1 hour, and then transferred to polyvinylidene fluoride (PVDF; Millipore, Etobicoke, Canada) membranes at 70 V for 1 hour. Membranes were blocked with 5% non-fat powdered milk in PBS (10 mM, pH 7.4) for 1 hour at 4°C, then incubated in primary, phospho-specific antibody (phospho-Stat3 Tyr705, 1∶1000, and phospho-Jak2 Tyr1007/1008, 1∶500, Cell Signaling Technology, Boston, USA) overnight in 5% bovine serum albumin for cytosolic fractions and 5% non-fat dry milk in PBS for nuclear STAT3 blots (BSA, Santa Cruz Biotechnology, Santa Cruz, USA) at 4°C. After multiple washes, blots were incubated in goat anti-rabbit HRP (1∶50,000; Abcam Inc., Cambridge USA) in 5% BSA for 90 minutes at room temperature. After multiple washes, proteins were detected with ECL (SuperSignal West Dura; Thermo Fisher Scientific, Rockford, USA) using FluorChem SP (Alpha Innotech Corporation, San Leandro, USA). After detection of phosphorylated proteins, blots were washed and stripped with Restore Western Blot Stripping Buffer (Thermo Fisher Scientific, Rockford, USA) for 20 minutes at room temperature. Membranes were washed and re-probed with total-specific antibodies (Stat3, 1∶1000, and Jak2 D2E12 rabbit mAb, 1∶500, Cell Signaling Techonlogy, Boston, USA) in the same manner as phospho-specific protein detection. Following total protein detection, ponceau staining confirmed equal loading. Protein bands corresponding to the predicted molecular weight of Jak2 (∼125 kDa) and Stat3 (∼86 kDa) were quantified using the AlphaEase FC Software, Version 5.0.2 (Alpha Innotech Corporation, San Leandro, USA) with background correction. As we were interested in determining the changes in the amount of activated protein, the ratio of phosphorylated to total protein was determined.

### Immunohistochemistry

7 µm muscle cross-sections were stained with antibodies against Pax7 (neat; cell supernatant from cells obtained from the DSHB, Iowa City USA); IL-6 (500 ng/mL, MAB 2061, R&D Systems, Minneapolis USA); p-STAT3 (1∶100, Cell Signaling Technologies Inc., Danvers USA) and laminin (1∶1000, L8271, Sigma-Aldrich, Oakville Canada). Secondary antibodies used were: Pax7 (AlexaFluor 488 or AlexaFluor 594, 1∶500, Invitrogen, Molecular Probes Inc., Camarillo USA or when using two mouse primary antibodies an immunoglobulin biotinylated secondary antibody, 1∶200, Dako Inc., Mississauga Canada; followed by a streptavidin-FITC fluorochrome, 1∶100, Carlsbad Biosource. USA); and Laminin (AlexaFluor 594, 1∶500, Invitrogen, Molecular Probes Inc., Camarillo USA). Histochemical methods were adapted from previously published methods from our lab [50], [17], [18], [20]. Briefly, for co-immunofluorescent staining (Pax7 and IL-6, Pax7 and Laminin), sections were fixed with 2% paraformaldehyde (PFA, Sigma-Aldrich, Oakville Canada) for 10 min followed by several washes in PBS. Sections were then covered for 60 min in a blocking solution containing, 2% BSA, 5% FBS, 0.2% Triton-X 100, 0.1% sodium azide. Following blocking, sections were incubated in the primary antibody at 4°C overnight. After several washes, sections were then incubated in the appropriate secondary antibodies. Sections were then re-fixed in 2% PFA (Sigma-Aldrich, Oakville Canada) to prevent migration of the secondary antibodies and re-blocked in 10% GS in 0.01% Triton-X 100 (Sigma-Aldrich, Oakville Canada). The sections were then incubated in the second primary antibody, followed by incubation in the appropriate secondary antibody. Sections were then washed with PBS and 4′,6-diamidino-2-phenylindole (DAPI, 1∶20000) (Sigma-Aldrich, Oakville Canada) for nuclear staining. Staining was verified using the appropriate positive and negative controls to ensure specificity of staining. Stained slides were viewed with the Nikon Eclipse 90*i* Microscope (Nikon Instruments, Inc., Melville USA) and images were captured and analyzed using the Nikon NIS Elements 3.0 software (Nikon Instruments, Inc., Melville USA). For cMyc immunodetection, serial sections were used to visualize both Pax7 (with Laminin) and cMyc. A secondary-only control image has been included to demonstrate the specificity of the cMyc antibody (Figure S1f). Pax7/Laminin staining was performed as described above, and cMyc was stained as follows: slides were dried and fixed in acetone for 10 minutes. Slides were then washed several times in 1× PBS followed by quenching of endogenous peroxidases for 30 minutes using 0.3% H_2_O_2_ solution. After washing, sections were then blocked in 10% goat serum in a 0.2% Triton-X 100 (Sigma-Aldrich, Oakville Canada) solution for 30 minutes. cMyc antibody (cell supernatant, DSHB, Iowa City USA) was used at 1∶2 in a 1% BSA solution and was then incubated for 2 hours at room temperature. Slides were then washed several times in PBST. The secondary IGB goat anti-mouse (DAKO Inc., Mississauga Canada) was incubated at 1∶200 for an hour followed by incubation in the Vectastain Elite ABC kit (Vector Laboratories, Burlington Canada) according to the manufacturer's instructions for 30 minutes. Following several washes in PBST, slides were developed using the DAB kit (Vector Laboratories, Burlington Canada) according to the manufacturer's instructions. Slides were counterstained using Mayer's hematoxylin (Sigma-Aldrich, Oakville Canada).

### Immunohistochemical Analysis

Immunohistochemical quantification and enumeration was performed at all time points (PRE, T1, T3, T24) for IL6^+^/Pax7^+^ (n = 12) and pSTAT3^+^/Pax7^+^ (n = 9) and images were taken at 40× magnification. For cMyc^+^/Pax7^+^ stain 7 subjects were analysed (n = 7) at PRE and T24 with images being taken at 20× magnification using full muscle cross-section stitched images. Two separate blinded reviewers quantified the co-localization of Pax7 and cMyc. Slides were viewed and images captured with the Nikon Eclipse 90*i* Microscope (Nikon Instruments, Inc., Melville USA) and Nikon NIS Elements 3.0 software (Nikon Instruments, Inc., Melville USA).

### Immunohistochemical quantification

Satellite cells were enumerated via double labelling with an anti-Pax7 antibody and DAPI. At least 300 myonuclei per timepoint were counted. Only those cells that were co-positive were counted as satellite cells. Furthermore, only cells associated with individual myofibers, that were not in the interstitial space, were counted as positive. IL6^+^/Pax7^+^ cells were only counted as positive if they were triple immunolabeled with DAPI and antibodies against IL-6 and Pax7. pSTAT3^+^/Pax7^+^ cells were only counted as positive if they were triple immunolabeled with DAPI and antibodies against pSTAT3 and Pax7. cMyc^+^/Pax7^+^ cells were only counted as positive if they were triple immunolabeled with Mayers Hemotoxylin and antibodies again cMyc and Pax7 with Laminin defining the SC niche. Non-fiber associated nuclei (interstitial nuclei) were not included in the quantification. All data are represented as a percentage of Pax7^+^ cells.

### Statistical Analysis

Statistical analysis and graphing were performed using Sigmstat 3.1.0 analysis software (Systat, SPSS Inc., San Jose USA) and Prism5 for Windows - version 5.01 (GraphPad Software Inc., La Jolla USA). mRNA, protein, IL-6 and creatine kinase plasma concentrations, IL-6^+^/Pax7^+^ and pSTAT^+^/Pax7^+^ enumeration were analysed using a 1-way repeated measures analysis of variance (ANOVA). cMyc^+^/Pax7^+^ enumeration was analysed via a two-tailed T-test. Statistical significance was set at P<0.05. Tukey's HSD post hoc test was used to analyse main effects and significant interactions. Results are presented as mean ±SEM.

## Supporting Information

Figure S1 (**S1a**) Pearson correlation between the serum concentrations of IL-6 (pg/mL) and cMyc (U/L); R^2^ = 0.3055; p<0.001. The correlation is representative of the individual data points presented as mean values ± SD (error bars). (**S1b**) Representative image of nuclear and cytoplasmic preparations with the cytoplasmic marker LDH present only in the cytoplasm and the nuclear marker p84 present only in the nuclear fraction. (**S1c**) Pearson correlation between the mRNA regulation (fold change) of IL-6 and SOCS3; R^2^ = 0.5984, p<0.001. The correlation is representative of the individual data points presented as mean values ± SD (error bars). (**S1d**) Pearson correlation between the mRNA regulation (fold change) of IL-6 and cMyc; R^2^ = 0.2876, p<0.001. The correlation is representative of the individual data points presented as mean values ± SD (error bars). (**S1e**) Pearson correlation between the mRNA regulation (fold change) of SOCS3 and cMyc; R^2^ = 0.5406, p<0.001. The correlation is representative of the individual data points presented as mean values ± SD (error bars). (**S1f**) Representative image of a muscle cross section stained with only the secondary and hematoxylin during the cMyc staining protocol. Note that there is no brown colour change indicating no false positivity caused from the addition of the secondary antibody.
(TIF)

Table S1 mRNA species that were analysed with their forward and reverse sequences, cDNA (ng) concentration used and annealing temperature (°C).
(DOC)
