# Supplementary figures and images for: IL-6 Induced STAT3 Signalling Is Associated with the Proliferation of Human Muscle Satellite Cells Following Acute Muscle Damage
Source: PLoS One. 2011 Mar 9;6(3):e17392. doi: 10.1371/journal.pone.0017392 (PMC3052298; doi:10.1371/journal.pone.0017392)

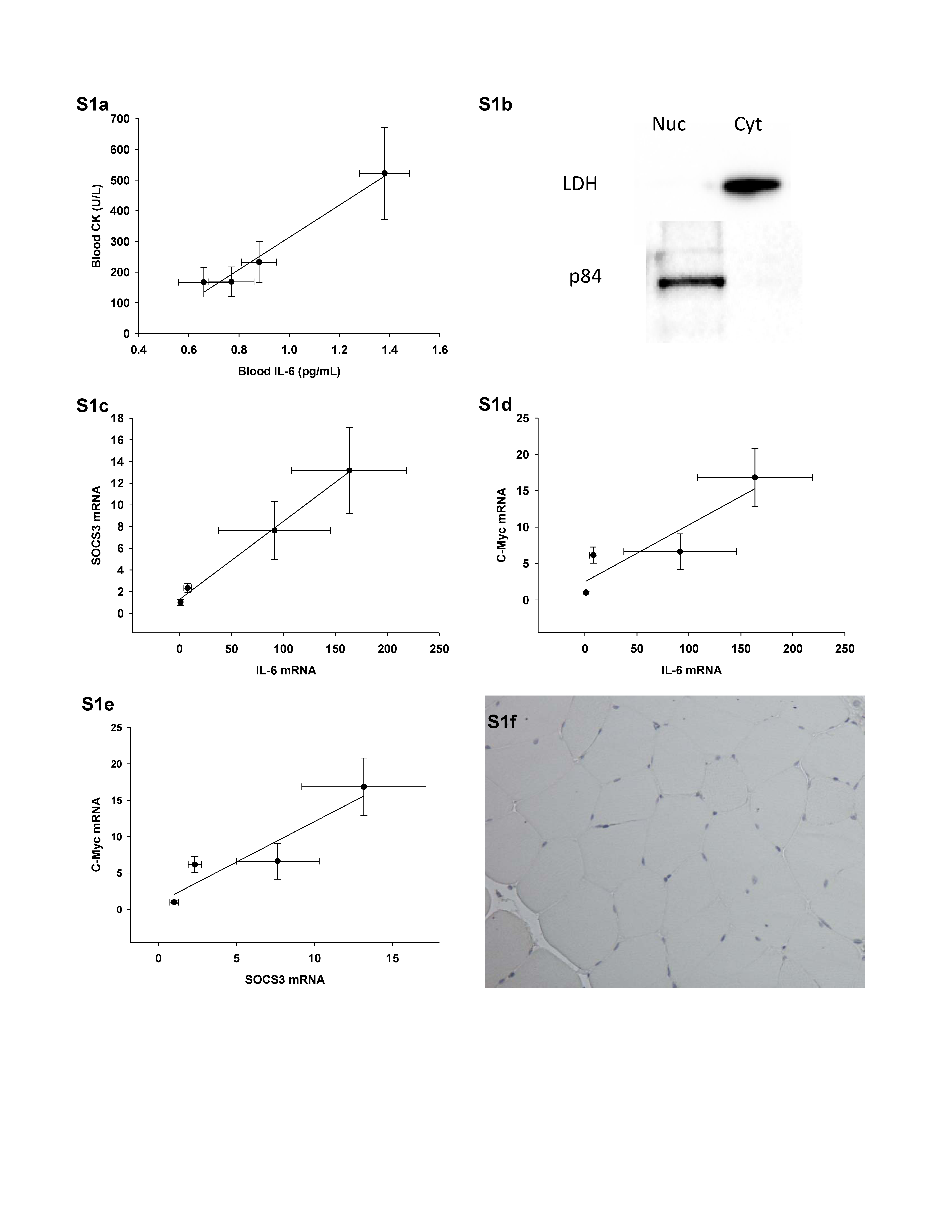

Supplement: Figure S1 — (S1a) Pearson correlation between the serum concentrations of IL-6 (pg/mL) and cMyc (U/L); R2 = 0.3055; p<0.001. The correlation is representative of the individual data points presented as mean values ± SD (error bars). (S1b) Representative image of nuclear and cytoplasmic preparations with the cytoplasmic marker LDH present only in the cytoplasm and the nuclear marker p84 present only in the nuclear fraction. (S1c) Pearson correlation between the mRNA regulation (fold change) of IL-6 and SOCS3; R2 = 0.5984, p<0.001. The correlation is representative of the individual data points presented as mean values ± SD (error bars). (S1d) Pearson correlation between the mRNA regulation (fold change) of IL-6 and cMyc; R2 = 0.2876, p<0.001. The correlation is representative of the individual data points presented as mean values ± SD (error bars). (S1e) Pearson correlation between the mRNA regulation (fold change) of SOCS3 and cMyc; R2 = 0.5406, p<0.001. The correlation is representative of the individual data points presented as mean values ± SD (error bars). (S1f) Representative image of a muscle cross section stained with only the secondary and hematoxylin during the cMyc staining protocol. Note that there is no brown colour change indicating no false positivity caused from the addition of the secondary antibody. (TIF) [file pone.0017392.s001.tif]
