# Supplementary material for: IL-6 Induced STAT3 Signalling Is Associated with the Proliferation of Human Muscle Satellite Cells Following Acute Muscle Damage
Source: PLoS One. 2011 Mar 9;6(3):e17392. doi: 10.1371/journal.pone.0017392 (PMC3052298; doi:10.1371/journal.pone.0017392)
Supplement: Table S1 — mRNA species that were analysed with their forward and reverse sequences, cDNA (ng) concentration used and annealing temperature (°C). (DOC) [file pone.0017392.s002.doc]

**Table S1**

| Gene | Forward Sequence | Reverse Sequence | Annealing Temperature (°C) |
| --- | --- | --- | --- |
| IL-6 | GAAAGCAGCAAAGAGGCACT | AGCTCTGGCTTGTTCCTCA | 62 |
| IL-6Rα | GACAATGCCACTGTTCACTG | GCTAACTGGCAGGAGAACTT | 60 |
| GP130 | AGAGTGGGACCAACTTCCTG | CCTTCCCACCTTCATCTGTG | 60 |
| SOCS3 | GACCAGCGCCACTTCTTCA | CTGGATGCGCAGGTTCTTG | 60 |
| Myf5 | ATGGACGTGATGGATGGCTG | GCGGCACAAACTCGTCCCCAA | 55 |
| c-Myc | CGTCTCCACACATCAGCACAA | TCTTGGCAGCAGGATAGTCCTT | 62 |
| MRF4 | CCCCTTCAGCTACAGACCCAA | CCCCCTGGAATGATCGGAAAC | 55 |
